# An Integrated Enzymatic Approach to Produce Pentyl Xylosides and Glucose/Xylose Laurate Esters From Wheat Bran

**DOI:** 10.3389/fbioe.2021.647442

**Published:** 2021-04-07

**Authors:** Chloé Jocquel, Murielle Muzard, Richard Plantier-Royon, Caroline Rémond

**Affiliations:** ^1^Université de Reims Champagne Ardenne, INRAE, FARE, UMR A 614, Chaire AFERE, Reims, France; ^2^Institut de Chimie Moléculaire de Reims, CNRS UMR 7312, Université de Reims Champagne-Ardenne, Reims, France

**Keywords:** biosurfactants, transglycosylation, (trans)esterification, biocatalysis, lignocellulose, agro-industrial co-products

## Abstract

Alkyl glycosides and sugars esters are non-ionic surfactants of interest for various applications (cosmetics, food, detergency,…). In the present study, xylans and cellulose from wheat bran were enzymatically converted into pentyl xylosides and glucose and xylose laurate monoesters. Transglycosylation reaction catalyzed by the commercial enzymatic cocktail Cellic Ctec2 in the presence of pentanol led to the synthesis of pentyl β-D-xylosides from DP1 to 3 with an overall yield of 520 mg/g of xylans present in wheat bran. Enzymatic hydrolysis of wheat bran with Cellic Ctec2 and subsequent acylation of the recovered D-glucose and D-xylose catalyzed by the commercial lipase N435 in the presence of lauric acid or methyl laurate produced one D-glucose laurate monoester and one D-xylose laurate monoester. An integrated approach combining transglycosylation and (trans)esterification reactions was successfully developed to produce both pentyl xylosides and D-glucose and D-xylose laurate esters from the same batch of wheat bran.

## Introduction

Surfactants include a large family of amphiphilic compounds exhibiting multiple properties as solubilizing agents, foaming agents, wetting agents, dispersants or emulsifiers depending on their composition and structure. Surfactants are thus widely used in the formulation of detergents, cosmetics, food and feed additives as well as pharmaceuticals. In the recent years, due to new regulatory context (REACH) and to the consumer demand for natural products, there has been a growing interest for biobased surfactants (Santos et al., 2016). Special attention is paid to non-ionic surfactants such as alkyl glycosides and sugar esters from renewable resources recognized as biodegradable and non-toxic for humans and the environment (von Rybinski and Hill, 1998; Neta et al., 2015). Alkyl glycosides, also called alkyl polyglycosides (APGs) due to the possible presence of a glycosidic part displaying a degree of polymerization superior to 1, are formed by a glycosidic polar head and an aliphatic chain on the anomeric position of the glycone moiety. APGs are generally synthesized from fatty alcohols and D-glucose *via* a Fischer glycosylation reaction (von Rybinski and Hill, 1998). They can display various aliphatic chains (butyl to tetradecyl). Short aliphatic chains lead to hydrotropic properties which can be useful for detergency or as foam boosters in cosmetics (von Rybinski and Hill, 1998; Bouxin et al., 2010). These surfactants are worldwide produced at a scale of 100,000–200,000 tons per year (Jerome et al., 2018). Sugar esters are formed by a polar glycosidic head associated with one or more hydroxyl group esterified by a fatty acid. The most common glycone polar heads are sorbitol, saccharose, and glucose while the aglycone part corresponds to long aliphatic chains derived from fatty acids or esterified fatty acids (Neta et al., 2015). Chemical routes requiring the use of polar solvents, a catalyst and high reaction temperatures are used for the formation of fatty acid esters by esterification or transesterification reactions (Polat and Linhardt, 2001). These chemical syntheses generally lead to complex mixtures of mono-esters, di- and higher esters and to different regioisomers.

Both APGs and sugar esters can be synthesized *via* biocatalytic routes involving some glycoside hydrolases (EC 3.2.1.X) and lipases (EC 3.1.1.3), respectively, which catalyze transglycosylation or acylation reactions (van Rantwijk et al., 1999; Adlercreutz, 2017). The high substrate specificity of enzymes allows the production of pure anomeric APG and regioselective sugar esters. Furthermore, the enzymatic reactions occur in a single step under mild reaction conditions (temperature, pressure, pH) which limits the energy and chemical requirements as well as the production of unwanted by-products. Enzymes are biocompatible, biodegradable, and most are non-hazardous. Compared to the rare metal catalysts required for several catalytic routes, most enzymes are produced by microbial fermentation processes in the presence of inexpensive and available renewable resources (Sheldon, 2020).

The development of biorefineries implies the valorization of largely available and inexpensive lignocellulosic agricultural, forestry and agro-industrial co-products and wastes into a wide range of molecules, materials and energies (Bozell, 2008; FitzPatrick et al., 2010). Among these co-products, wheat bran is a feedstock of interest for biorefineries. Taking into account that all produced wheat for human consumption is milled, the worldwide production of wheat bran represent almost 150 million tons per year (Pruckler et al., 2014). This lignocellulosic biomass contains a high rate of cellulose, xylans, starch and proteins whereas the lignin content is low (Apprich et al., 2014).

The objective of our study was to produce pentyl xylosides and D-glucose and D-xylose laurate esters from non-pretreated wheat bran *via* biocatalytic routes. The strategy was to develop a transglycosylation reaction with the xylanase activity from the commercial enzymatic CellicCtec2 cocktail to access pentyl xylosides directly from wheat bran. The production of D-glucose and D-xylose laurate esters was achieved by a two-step biocatalytic strategy. In the first step, wheat bran was hydrolyzed with cellulase and xylanase activities from CellicCtec2 to recover D-glucose and D-xylose monosaccharides which were further (trans)esterified with methyl laurate or lauric acid in the presence of the commercial lipase N435. Finally, an integrated approach combining enzymatic transglycosylation and (trans)esterification has been developed to produce both pentyl xylosides and D-glucose and D-xylose laurate esters from the same batch of non-pretreated wheat bran.

## Materials and Methods

### Materials

Wheat bran (WB) (0.5–2 mm) was a generous gift by the company BioWanze (Wanze, Belgium).

Acetonitrile (>99.9%) was purchased from Carlo Erba Reagents (Dasit Group S.p.A, Cornaredo, Italy). 2-methylbutan-2-ol (2M2B, 99%), pentan-1-ol (>99%), lauric acid (99%), methyl laurate (99%), molecular sieves (3 and 5 Å) and the enzyme Novozym 435 (immobilized lipase on acrylic resin from *Candida antarctica*) were purchased from Sigma-Aldrich Corp. (St. Louis, United States). CellicCtec2 enzyme was a generous gift from Novozymes.

### Synthesis of Pentyl Xylosides

Transglycosylation reactions were catalyzed by the enzyme Cellic Ctec2 (50 xylanase IU/mL) in the presence of WB (5%, w/v) and 50% pentanol in water (v/v) at 50°C during 48 h with magnetic stirring (400 rpm). Reactions were performed in triplicates in 1 mL final volume. To prepare sufficient quantities of pentyl xylosides, an up-scaling of the reaction was carried out with 400 mL as final volume. Pentyl xylosides were recovered by separating the two pentanol and aqueous phases at the end of the reaction, pentyl xylosides being present in the pentanol phase.

Transglycosylation reactions (1 mL) were carried out under the same reaction conditions in the presence of Avicel cellulose (2%, w/v) and 50% pentanol in water (v/v). At the end of the reactions, samples were boiled during 10 min and centrifuged during 10 min at 5,000 g. Analyses were carried out from the recovered aqueous and pentanol phases.

### Wheat Bran Hydrolysis

Wheat bran (5%, w/v in water) was hydrolyzed by Cellic Ctec2 at 15 FPU/g of bran during 72 h at 50°C with magnetic stirring (400 rpm). Reaction was conducted into a 500 mL reaction volume.

### Synthesis of Sugar Laurate Esters

D-glucose and D-xylose laurate esters were synthesized using 1% lipase N435 from Novozymes in 2M2B in the presence of lauric acid or methyl laurate at 100 or 300 mM. After lyophilization, the wheat bran hydrolysate (see section “Wheat Bran Hydrolysis”) or the aqueous phase recovered from transglycosylation reaction (see section “Synthesis of Pentyl Xylosides”) was added in the acylation reaction mixture in order to obtain a total D-glucose and D-xylose concentration of 100 mM. Molecular sieves 3 Å or 5 Å were present at 10% (w/v), respectively, for reactions conducted with lauric acid or methyl laurate. Reactions (5 mL) were carried out in triplicates at 50°C under magnetic stirring (400 rpm) during 48 h.

### Analyses

#### Xylanase and Cellulase Activities

Xylanase and cellulase activities from the Cellic Ctec2 cocktail were measured as previously reported in the presence of beechwood xylans (Roth) and Whatman cellulose paper 1 CHR (Dutscher) (Rémond et al., 2010).

#### TLC Analysis

TLC analyses were performed in order to visualize transglycosylated products using Kieselgel 60 F_254_ aluminum-backed sheets (Merck) and ethyl acetate/acetic acid/H_2_O: 7/2/2 as the mobile phase. Transglycosylated products were revealed using 0.2% (v/v) orcinol in H_2_SO_4_ (20%, v/v).

Pentyl xylosides with DP1, 2, and 3 produced as previously described (Ochs et al., 2011) were used as standards.

#### Sugar Quantification

The sugar content of WB was determined by HPAEC-PAD, High Performance Anion Exchange Chromatography coupled with Pulsed Amperometry Detection with a Dionex CarboPac PA-1 column (4 × 250 mm, Thermo Fisher Scientific) after acid hydrolysis (Rémond et al., 2010). D-Glucose, D-xylose and L-arabinose are the main monosaccharides present with, respectively, 30.5 ± 1.0, 18.4 ± 0.3, and 13.1 ± 0.1% of dry matter (DM). The content of D-galactose is much lower (1.1 ± 0.02% DM). Starch content, determined with the total starch HK assay kit (Megazymes), corresponds to 11.6 ± 0.4% DM.

D-glucose and D-xylose released during the hydrolysis of WB with Cellic Ctec2 as well as the monosaccharides present within the aqueous phase from the transglycosylation reactions were quantified by HPAEC-PAD (ICS 5000, Thermo Fisher Scientific, Courtaboeuf, France) after injection on a Dionex CarboPac PA-1 column according to a previously described method (Rémond et al., 2010).

#### Pentyl Xylosides and Glucose Esters Quantification

Pentyl xylosides were quantified by HPLC in both aqueous and pentanol phases recovered from transglycosylation reactions as previously described (Ochs et al., 2011). The quantification was performed by HPLC using a RP-C18 column (Nucleoshell RP 18, 250 × 4.6 mm, Macherey Nagel). Standard alkyl xylosides were purified as described previously (Ochs et al., 2011). Products were eluted at 0.6 mL/min with a mobile phase composed of an acetonitrile:water mixture (20:80). The detection of eluates was performed with a dynamic light scattering detector (ELSD-LT II, Shimadzu). Pentyl β-D-xylosides DP 1–3 used as standards were produced and purified as previously described (Ochs et al., 2011).

D-glucose and D-xylose laurate esters were quantified by HPLC as reported in a previous study (Meline et al., 2018). Briefly, the quantification was performed by HPLC (Prominence, Shimadzu) using a Nucleoshell^®^ RP 18 plus 5 μm, 250 × 4.6 mm (Macherey Nagel) column at 40°C. D-Glucose and D-xylose laurate esters were eluted at 0.8 mL/min, at 40°C and with a 80:20 acetonitrile : water mobile phase. The detection was performed with a dynamic light scattering detector (ELSD-LT II, Shimadzu) at 40°C under 350 kPa azote pressure. Pure D-xylose laurate monoesters, diesters (Meline et al., 2018) and pure D-Glucose laurate monoester (Martinez-Garcia et al., 2021) were prepared and characterized as previously described and were used as standards.

## Results and Discussion

### Synthesis of Pentyl β-D-Xylosides

**FIGURE 1:**
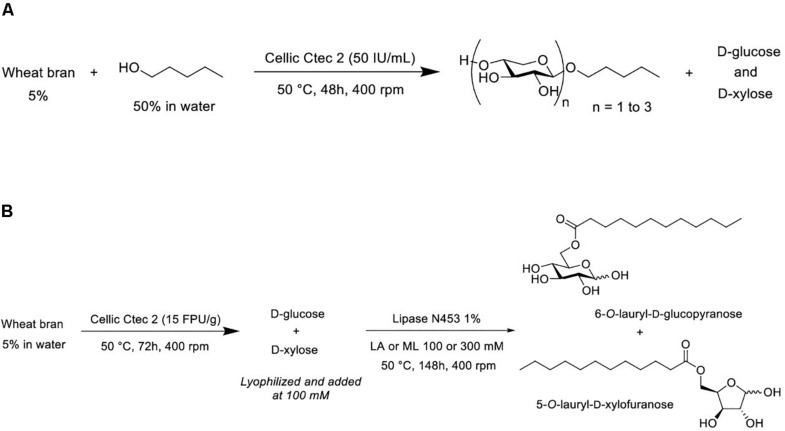
**(A)** Transglycosylation reaction to produce pentyl β-D xylosides from wheat bran. **(B)** Sequential hydrolysis and (trans)-esterification reactions to produce sugar esters. LA, lauric acid; ML, methyl laurate.

**FIGURE 2:**
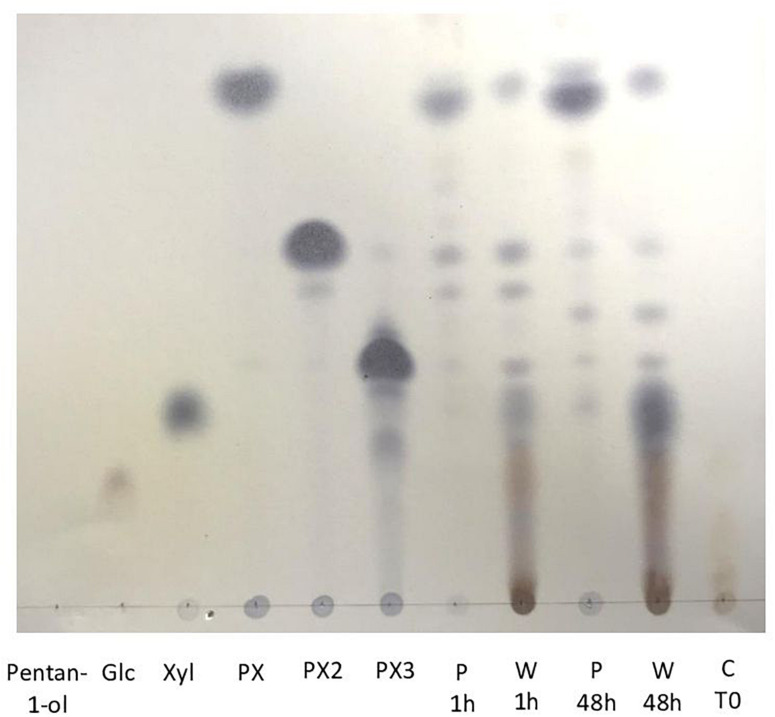
TLC analysis of pentyl β-D xylosides produced from transglycosylation reaction catalyzed by Cellic Ctec2 in presence of wheat bran and pentanol 50% in water (v/v) during 48 h at 50°C. Pentanol, glucose, xylose, pentyl xyloside DP1 (PX) pentyl xyloside DP2 (PX2) and pentyl xyloside DP3 (PX3) were used as standards. Aqueous (W) and pentanol (P) phases were recovered after 1 and 48 h of the transglycosylation reactions. C T0 corresponds to a control transglycosylation reaction at time zero.

As described in Figure 1A, transglycosylation reaction was catalyzed by the enzymatic cocktail CellicCtec2 (50 IU xylanase/mL) in the presence of WB (5%, w/v) and pentanol at 50°C during 48 h with magnetic stirring. Aliquots were taken after 1 and 48 h of reaction. Pentanol was chosen as the acceptor in the reaction in order to produce short chain APG with hydrotropic properties. Cellic Ctec2 is a commercial enzymatic cocktail displaying a high xylanase activity (11,000 IU/mL) as well as an important cellulase activity (100 FPU/mL). TLC analysis allowed the visualization of pentyl xylosides from DP1 to 3 indicating that the xylanase activity of Cellic Ctec2 was able to catalyze transglycosylation reactions (Figure 2). On the TLC, some minor spots different from the pentyl xylosides DP1, DP2, and DP3 are present and could probably be attributed to the presence of pentyl xylosides substituted with arabinose group. The minor spot present between DP2 and DP3 pentyl xylosides could correspond to pentyl [3′-*O*-α-L-arabinofuranosyl]-β-D-xylobioside that was produced and described in our previous work dealing with the transglycosylation reaction with arabinoxylans from oat spelt (Ochs et al., 2011). In order to check if Cellic Ctec2 could catalyze transglucosylation reactions from cellulose, assays were carried out with Avicel^®^ cellulose (2% w/v) and pentanol under the same reaction conditions. Whereas glucose was detected indicating that a hydrolysis reaction had occurred, no pentyl glucoside was synthezised confirming that Cellic Ctec2 did not catalyze a transglycosylation reaction from cellulose (data not presented).

**FIGURE 3:**
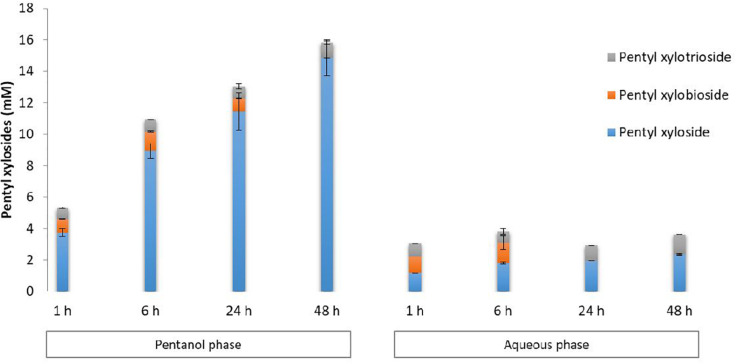
HPLC quantification of pentyl β-D xylosides from DP1–3 in pentanol and aqueous phases recovered from transglycosylation reactions performed with Cellic Ctec2 in the presence of WB and pentanol in aqueous media. Error bars were obtained from triplicate reactions.

Pentyl β-D-xylosides were quantified by HPLC in both aqueous and pentanol phases as reported in Figure 3. Pentyl xyloside DP1 was the major product and was mainly detected in the pentanol phase. Its concentration increased according to the duration of the reaction to reach 14.9 mM after 48 h reaction. In the aqueous phase, pentyl xyloside DP1 was also present in a lower extent indicating a lower miscibility in water compared to pentanol. The maximal concentration for pentyl xyloside DP1 in the water phase reached 2.3 mM after 48 h reaction. Pentyl xylosides DP2 and DP3 were detected with a similar content in both pentanol and aqueous phases. The maximal concentrations for pentyl xylobioside DP2 were obtained after 6 h of reaction with 1.2 and 1.3 mM, respectively, in the pentanol and aqueous phases. At 48 h, no pentyl xylobioside DP2 was detected in both phases whereas pentyl xylotrioside DP3 was still present (1.0 and 1.3 mM, respectively, in pentanol and aqueous phases) indicating that a secondary hydrolysis was taking place more rapidly with pentyl xylobioside than with pentyl xylotrioside.

The concentration of β-D-xylosides from DP1 to 3 corresponded to 19.44 mM mainly in pentanol phase after 48 h of transglycosylation reaction (Table 1) so about 520 mg of pentyl xylosides were produced per gram of xylans present within WB in both pentanol and aqueous phases at the end of the reaction.

**TABLE 1:** Quantification of total β-D-pentyl xylosides (DP1–DP3) and monosaccharides present in the pentanol and aqueous phases after 48 h of transglycosylation reaction catalyzed by Cellic Ctec2 in the presence of WB and pentanol 50% in water.

	**Pentanol phase**	**Aqueous phase**	**Total in pentanol and aqueous phases**
Total pentyl xylosides concentration (mM)	15.82	3.63	19.44
Yields of transglycosylation (g/g xylans in WB)	0.40	0.12	0.52
Average DP	1.12	1.66	1.28
D-glucose concentration (mM)	0	72.2	72.2
D-xylose concentration (mM)	0	36.7	36.7

The enzymatic synthesis of alkyl xylosides has been largely described with various xylanases that led to various yields of synthesis according to the xylans sources, acceptors types and reaction conditions. Whereas most transxylosylation reactions were developed with xylans as donors, there are few examples describing the synthesis of alkyl glycosides directly from a lignocellulosic feedstock. Matsumura et al. (1999) used a xylanase

from *Aureobasidium pullulans* for the transxylosylation of xylans with octan-1-ol and 2-ethylhexanol. Xylopyranosides with DP 1 and 2 were produced from xylans and 2-ethylhexanol with a maximal yield equivalent to 110 and 54 mg/g of xylans, respectively, after 12 h of catalysis. The authors indicated that similar results were obtained with octan-1-ol. In another study, the same xylanase was tested for the transxylosylation of xylans and octan-1-ol in supercritical carbon dioxide and fluoroform fluids (Nakamura et al., 2000). The production of total octyl β-D-xylosides (DP1–3) reached 250 mg/g of xylans. A xylanase from *Thermotoga neapolitana* was able to produce 13.2, 8.7, and 2.8 mM of hexyl β-D-xyloside with DP 1, 2, and 3 after 3 h of transxylosylation reaction in presence of birchwood xylans and hexan-1-ol (Tramice et al., 2007). In a previous study, we compared the synthesis of pentyl β-D-xylosides from birchwood xylans and pentan-1-ol catalyzed by a xylanase from *Thermobacillus xylanilyticus* and a commercial xylanase (Novozymes NS 50030). In the best conditions the total yields were, respectively, 857 and 714 mg/g xylan and the average DP was 1.8 and 2.1, respectively, for the reaction with the *T. xylanilyticus* xylanase and with the NS-50030 xylanase (Ochs et al., 2011). The xylanase from *T. xylanilyticus* was also tested for the production of pentyl β-D-xylosides by using as donors a mixture of xylo-oligosaccharides recovered from a hydrothermally treated wheat bran (Ochs et al., 2011). The total yield reached 222 mg/g equivalent xylans and the average DP was 1.9. However, the direct transxylosylation of wheat bran with this xylanase in presence of octan-1-ol rather inefficient with a yield of octyl xylosides production equivalent to 3.5 mg/g xylan present in wheat bran (Ochs et al., 2011). In the present study, we demonstrated for the first time that the synthesis of alkyl β-D-xylosides and notably pentyl xylosides can be achieved directly from a lignocellulosic biomass without any pretreatment and our results indicate that the CellicCtec2 enzyme catalyzed the tranxylosylation reaction with an important level of efficiency as yields obtained were in the same range as those described in the literature when xylans are used as donors.

The average DP of pentyl xylosides is 1.12 and 1.66 in pentanol and aqueous phases, respectively. This result can be explained by the greater solubility of pentyl β-D-xylosides in the aqueous phase when the DP of the glycone part is increased.

During the transglycosylation reaction, water acts as a competing nucleophile and causes primary hydrolysis of xylans and secondary hydrolysis of transglycosylated products (Mackenzie et al., 1998). Competitive hydrolysis during the transglycosylation reaction with pentanol led to the release of D-glucose (72.2 mM) and D-xylose (36.7 mM) which were detected in the aqueous phase (Table 1). In the literature data, various strategies were developed to promote transglycosylation reactions over hydrolysis. Decreasing the water activity was reported to favor the transglycosylation catalyzed by a glucosidase in presence of hexanol and activated glycosides (Hansson et al., 2001). Some protein engineering approaches highlighted the role of some aminoacid residues of the catalytic sites of glycoside hydrolases for the transglycosylation/hydrolysis ratio (Matsui et al., 1994; Hansson and Adlercreutz, 2001; Arab-Jaziri et al., 2013; Lundemo et al., 2017). Recently, the mutation of a key residue (W126) from the aglycone subsite of a xylanase, identified by a modeling approach, into alanine was reported to enhance the transxylosylation of the mutated enzyme in the presence of xylans and pentanol (Brusa et al., 2018). In regards to the D-xylose equivalent present within WB, the % of xylose equivalent converted into pentyl xylosides reached 26.4% whereas the % of xylose equivalent released as a monosaccharide corresponded to 71.8% indicating that almost all the xylose content of WB was converted during the process (Table 2). In the present study, the competition between hydrolysis and transglycosylation was not considered as a bottleneck since the recovered monosaccharides from the reaction can be used as acyl acceptors for sugar esters synthesis (see section “An Integrated Approach for the Synthesis of Pentyl Xylosides and Sugar Laurate Esters From Wheat Bran”).

**TABLE 2:** % of xylose from WB converted into pentyl xylosides (DP1–DP3), into xylose, % of non-converted xylose after 48 h of transglycosylation reaction catalyzed by Cellic Ctec2 in the presence of WB and pentanol 50% in water.

**% of xylose equivalent from WB converted in pentyl xylosides** (from transglycosylation reaction)	26.4
**% of xylose equivalent from WB converted in xylose** (from hydrolysis reaction)	71.8
**% of xylose equivalent from WB non-converted**	1.8

**% are expressed as equivalent xylose initially present in WB.**

### Synthesis of Sugars Laurate Esters

The synthesis of sugar laurate esters was achieved in a two-step process as described in Figure 1B. The first step corresponds to the enzymatic hydrolysis of WB with Cellic Ctec2 to produce a hydrolysate containing mainly both D-glucose and D-xylose. In a second step, the mixture with the two monosaccharides was acylated with lauric acid or methyl laurate in the presence of commercially available immobilized lipase N435.

Hydrolysis of WB performed with Cellic Ctec2 at 15 FPU/g during 72 h led to the release of 25.4 mM of D-glucose and 43.0 mM of D-xylose, respectively, due to the cellulase and xylanase activities within the enzymatic cocktail. This corresponds to 30 and 70% of glucose and xylose, respectively, released from WB by the Cellic Ctec2 cocktail under the hydrolysis conditions tested. The high xylanase activity in the enzymatic cocktail (11,000 IU xylanase/mL compared to 100 FPU/mL) could explain the greater release of xylose compared to glucose.

The acylation step was then carried out with the lyophilized hydrolysate solubilized in the reaction mixture to reach a total concentration of 100 mM for D-glucose and D-xylose (37 mM for D-glucose and 63 mM for D-xylose). Acylation with lipase N435 was studied with methyl laurate (transesterification) and lauric acid (esterification) at 100 and 300 mM.

**FIGURE 4:**
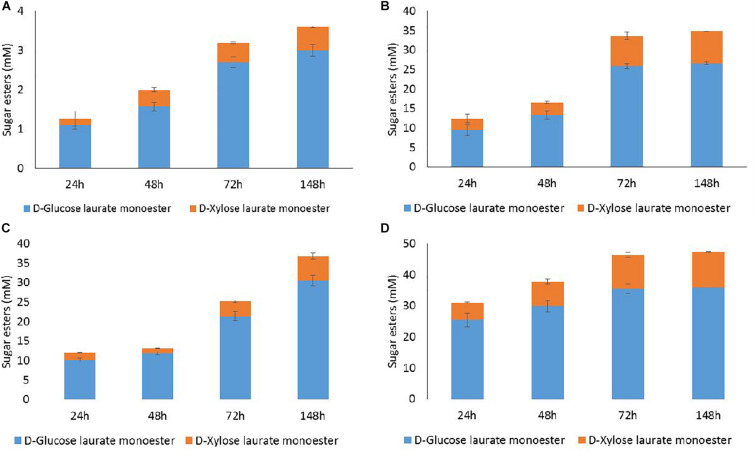
HPLC quantification of D-glucose and D-xylose laurate esters recovered from acylation reactions performed with the lipase N435 in 2M2B from WB hydrolysate at 100 mM (D-glucose + D-xylose) and lauric acid at 100 mM **(A)** or 300 mM **(B)**, methyl laurate at 100 mM **(C)** 300 mM **(D)**. Error bars were obtained from triplicate reactions.

Numerous data from literature indicated that acylation of D-glucose with fatty acids, using lipase N435, selectively occurred at the primary position leading to the 6-*O*-monoester of D-glucopyranose (Arcos et al., 1998; Cao et al., 1998). In a previous study, we demonstrated that acylation of pure D-xylose with vinyl laurate catalyzed by lipase N435 yielded a mixture of D-xylofuranose laurate esters: one major monoester (5-*O*-lauryl-D-xylofuranose), a minor monoester (4-*O*-lauryl-D-xylopyranose) and two diesters (2,5-di-*O*-lauryl-D-xylofuranose and 3,5-di-*O*-lauryl-D-xylofuranose) (Meline et al., 2018). Based on these data, HPLC quantification of 6-*O*-lauryl-D-glucopyranose as well as the two D-xylofuranose laurate monoesters and the two D-xylose laurate diesters was carried out. The present results indicate that lipase N435 catalyzed both esterification and transesterification reactions from lauric acid and methyl laurate as acyl donors, respectively (Figure 4). For all the conditions tested, 6-*O*-lauryl-D-glucopyranose was produced as the main laurate ester during both esterification and transesterification reactions. In the case of D-xylose, the monoester 5-*O*-lauryl-D-xylofuranose was produced and no D-xylofuranose diesters were detected. The esterification regioselectively took place onto the primary hydroxyl group for the furanose isomers of D-xylose. The higher acylation efficiency for D-glucose compared to D-xylose could probably be attributed to the low ratio of the furanose isomers of D-xylose in the reaction mixture (Drew et al., 1998). It could also be explained by a difference in dissolution of D-glucose and D-xylose in 2M2B as previously described for D-glucose and D-fructose in 2M2B (Engasser et al., 2008). Finally, despite the well-known versatility of the lipases N435 for various acyl acceptors (Ortiz et al., 2019), the lower efficiency of the lipase N435 for D-xylose acylation could reflect a lower affinity of the enzyme for D-xylose compared to its affinity for D-glucose. Similar results were obtained in a previous study describing the transesterification of a mixture of D-glucose and D-xylose with vinyl octanoate catalyzed by the lipase N435. It was demonstrated that the conversion of D-xylose was lower (<0.1% of D-xylose conversion) compared to D-glucose (0.7% conversion) (Siebenhaller et al., 2017).

Esterification with lauric acid 100 mM led to a low acylation rate with the synthesis of 3 mM of D-glucose laurate monoester and 0.6 mM of D-xylose laurate monoester at 148 h (Figure 4A). The extent of esterification increased with 300 mM lauric acid to the production of 26.6 mM of D-glucose laurate monoester and 8.2 mM of D-xylose laurate monoester after 148 h (Figure 4B). Under these conditions, the conversion rates were 72 and 13% for D-glucose and D-xylose, respectively, from WB hydrolysate. Levels of acylation were quite similar for 72 and 148 h in the presence of 300 mM lauric acid. Same trends but with higher acylation efficiency were observed in case of transesterification of D-glucose and D-xylose from WB hydrolysate with methyl laurate (Figures 4C,D). Maximal concentrations of D-glucose laurate monoester (36 mM) and D-xylose laurate monoester (11 mM) were obtained from 72 h with methyl laurate 300 mM corresponding to 97 and 17% of D-glucose and D-xylose conversion, respectively (Figure 4D).

The acylation of D-glucose with the lipase N435 has been extensively described and the yields of acylation were reported to depend on various parameters such as the nature and the concentration of the acyl donor and the type of solvent (Gumel et al., 2011a). In presence of a mixture of DMSO and *tert*-butanol, the lipase N435 esterified D-glucose with decanoic acid with a maximal yield equivalent to 65% (Gumel et al., 2011b). 6-*O*-β-D-Glucose fatty acid monoesters were synthesized from D-glucose and fatty acids or fatty acid methyl esters with various chain lengths (C8, C16, C18) with the lipase N435 (Yan et al., 1999). The highest yields (up to 90%) were achieved in ethyl methylketone or acetone as solvent. The acylation of D-glucose catalyzed by the lipase N435 with myristic acid into 2M2B as solvent allowed the synthesis of 6-*O*-myristyl-D-glucose with a yield equivalent to 45% (Cauglia and Canepa, 2008). The enzymatic acylation of D-xylose to produce xylose-based esters was less extensively studied compared to the acylation of hexoses. The lipase-catalyzed synthesis of fatty acid D-xylose esters was investigated but no information concerning neither the regioselectivity of the reaction nor the structures of the esters were available. The efficiency of different lipases to produce D-xylose-based esters was tested from 1,2-*O*-isopropylidene-α-D-xylofuranose as acyl acceptor in presence of various fatty acids (Ward et al., 1997). The lipase N435 was the most efficient and achieved a global conversion equal to 90%. In another study, this lipase produced a mixture of 5-*O*-lauryl-D-xylofuranose, 2,5-di-*O*-lauryl-D-xylofuranose and 3,5-di-*O*-lauryl-D-xylofuranose from D-xylose and vinyl laurate with a global conversion yield reaching 74.9% (Meline et al., 2018).

In our study, the enzymatic acylation was performed with a mixture of D-glucose and D-xylose that could explain the lower yield for D-xylose acylation. Regarding the D-glucose content of WB, 21.6 and 29.1% of D-glucose from WB were converted into laurate monoester in case of esterification and transesterification, respectively (Table 3). The conversion of D-xylose from WB reached 9.1 and 11.9%, respectively, in presence of lauric acid and methyl laurate (Table 3).

**TABLE 3:** % of glucose and xylose from WB converted into laurate esters catalyzed by the lipase N435 from glucose and xylose present in the hydrolysate (hydrolysis reaction) or in the transglycosylation phase (transglycosylation reaction) after 72 h of acylation reaction.

	**From the aqueous phase of WB hydrolysis**		**From the transglycosylation aqueous phase of WB hydrolysis**	
	**Esterification**	**Trans-esterification**	**Esterification**	**Trans-esterification**
% of D-glc equivalent converted	21.6	29.1	66.2	77.6
% of D-xyl equivalent converted	9.1	11.9	11.8	13.4

**Esterification or transesterification were conducted in the presence of lauric acid or methyl laurate (300 mM). % are expressed as equivalent glucose or xylose initially present in WB.**

### An Integrated Approach for the Synthesis of Pentyl Xylosides and Sugar Laurate Esters From Wheat Bran

**FIGURE 5:**
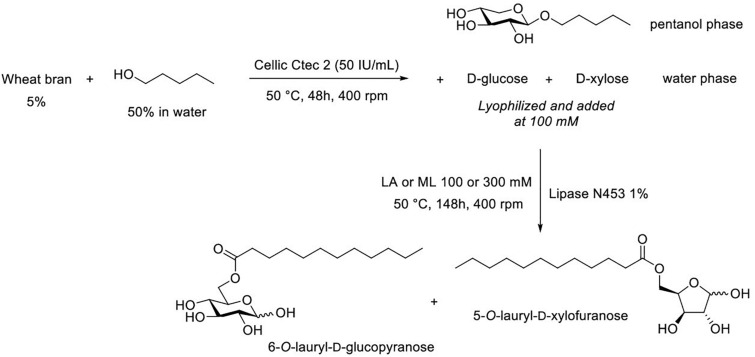
Successive transglycosylation and (trans)esterification reactions to produce pentyl xyloside and sugar esters from a single batch of wheat bran.

As described above, transglycosylation reaction catalyzed by Cellic Ctec2 from WB in the presence of pentanol led to the formation of pentyl xylosides but also to the production of both monosaccharides D-glucose and D-xylose in the aqueous phase. Monosaccharides from this latter phase could thus be used as acyl acceptors for the production of D-glucose and D-xylose laurate esters (Figure 5).

Although the hydrolysis and transglycosylation reactions were carried out with Cellic Ctec2, the D-glucose/D-xylose ratios were different in the hydrolysate (ratio = 0.6) and in the aqueous phase resulting from transglycosylation (ratio = 2). The lower amount of D-xylose in the aqueous transglycosylation phase can be attributed to the competitive transglycosylation and hydrolysis reactions which led to both pentyl xylosides synthesis and D-xylose release.

**FIGURE 6:**
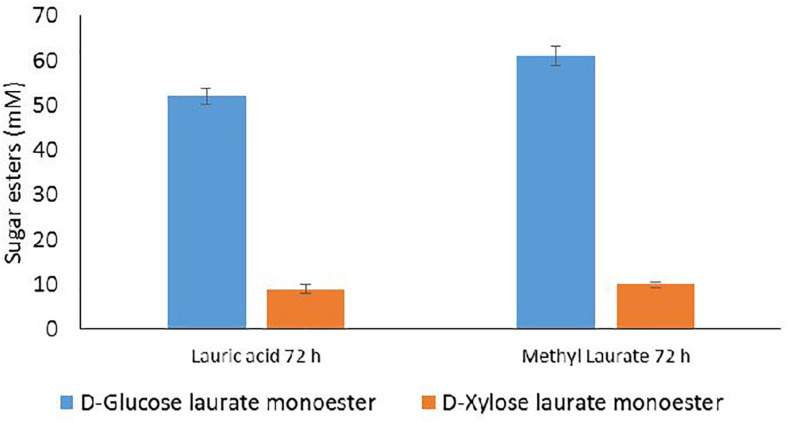
HPLC quantification of D-glucose and D-xylose laurate esters recovered from acylation reactions performed with the lipase N435 in the presence of 100 mM D-glucose and D-xylose recovered from the aqueous phase of transglycosylation reaction and lauric acid or methyl laurate at 300 mM in 2M2B during 72 h. Error bars were obtained from triplicate reactions.

The acylation reaction was performed after lyophilization of the aqueous transglycosylation phase and a subsequent solubilization in the reaction mixture in order to obtain a concentration of total sugars of 100 mM (66 mM D-glucose and 34 mM D-xylose). Acylation conditions were selected according to the best results obtained for the acylation of D-glucose and D-xylose mixtures from the WB hydrolysate (lauric acid or methyl laurate at 300 mM during 72 h). Results are presented in Figure 6.

As observed in the case of the acylation of D-glucose and D-xylose from WB hydrolysate, the esterification and transesterification reactions conducted with the aqueous transglycosylation phase mainly led to the synthesis of 6-*O*-lauryl-D-glucopyranose whereas 5-*O*-lauryl-D-xylofuranose was produced with a lower extent. The concentrations of D-glucose laurate monoester and D-xylose laurate monoester reached 52 and 9 mM, respectively, corresponding to 79 and 20.5% of D-glucose and D-xylose conversion in the presence of lauric acid as the acyl donor. Transesterification reaction with methyl laurate led to the production of 61 and 10 mM of D-glucose laurate monoester and D-xylose laurate monoester indicating that 92% of D-glucose and 23% D-xylose were converted during the reaction catalyzed by the lipase N435. The concentrations of D-glucose laurate monoester and D-xylose laurate monoester were thus higher in case of the acylation performed from the transglycosylation aqueous phase compared to the enzymatic acylation conducted from the hydrolysis reaction for which 36 and 11 mM of D-glucose laurate monoester and D-xylose laurate monoester were, respectively, synthesized. Furthermore, this integrated approach allowed the synthesis of both pentyl xylosides and sugar laurate esters from one unique WB batch whereas two WB batches were required for the separated process. The % of D-glucose conversion regarding the glucose content in WB was higher for the integrated approach compared to the separated one and reached 66.2 and 77.6%, respectively, for the esterification and transesterification reactions (Table 3). This can be probably explained by the higher concentration of D-glucose within the transglycosylation aqueous phase compared to the concentration in the hydrolysis phase. The % of D-xylose conversion from WB into laurate monoester was quite similar for both integrated and separated approach (Table 3).

## Conclusion

In the present study, two non-ionic biosurfactants families were enzymatically produced from wheat bran : alkyl xylosides and monosaccharide esters. The enzymatic processes allowed to produce pentyl xylosides and D-glucose and D-xylose laurate esters directly from wheat bran without any physico-chemical pretreatment of this raw material. A separated approach combining enzymatic transglycosylation, hydrolysis and (trans)esterification was first studied. An integrated process combining transglycosylation and (trans)esterification from one WB batch was also developed. This original integrated approach offers new possibilities for WB valorization into high added-value molecules.

## Data Availability Statement

The original contributions presented in the study are included in the article/Supplementary Material, further inquiries can be directed to the corresponding author/s.

## Author Contributions

CR, MM, and RP-R conceived and supervised the experiments. CJ performed the experiments and analyzed the results. CR wrote the manuscript. MM and RP-R revised the manuscript. All authors contributed to the article and approved the submitted version.

## Conflict of Interest

The authors declare that the research was conducted in the absence of any commercial or financial relationships that could be construed as a potential conflict of interest.
