# Supplementary material for: An Integrated Enzymatic Approach to Produce Pentyl Xylosides and Glucose/Xylose Laurate Esters From Wheat Bran
Source: Front Bioeng Biotechnol. 2021 Apr 7;9:647442. doi: 10.3389/fbioe.2021.647442 (PMC8058420; doi:10.3389/fbioe.2021.647442)

Supplementary material : HPLC chromatograms

Pentyl xylosides

The quantification was performed by HPLC (Prominence, Shimadzu) using a RP-C18 column (Nucleoshell RP 18, 250 × 4.6 mm, Macherey Nagel). Standard alkyl xylosides were purified as described previously (Ochs, Muzard et al. 2011). Products were eluted at 0.6 mL/min with a mobile phase composed of an acetonitrile:water mixture (20:80). The detection of eluates was performed with a dynamic light scattering detector (ELSD-LT II, Shimadzu).

1. Pentyl xyloside DP1 (standard) : Rt 10.7 min

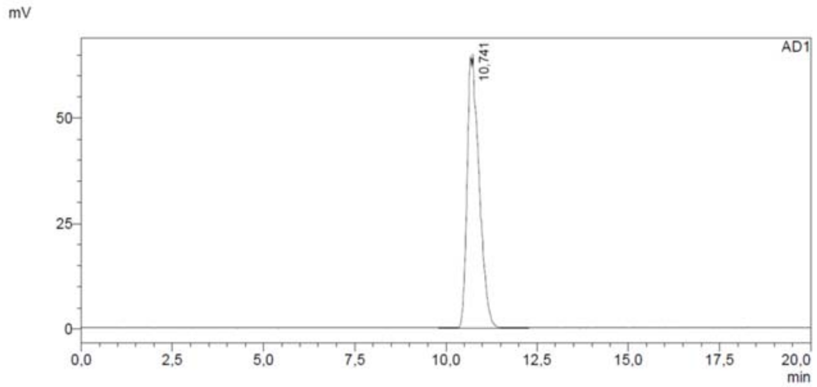

2. Pentyl xyloside DP2 (standard) : Rt 8.8 min

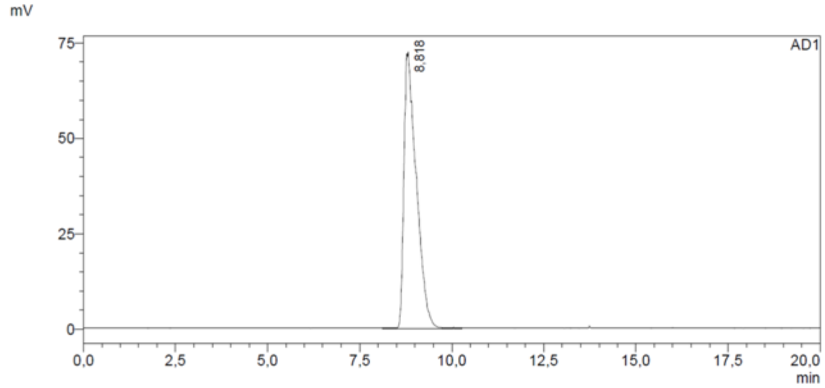

3. Pentyl xyloside DP3 (standard) : Rt 7.7 min

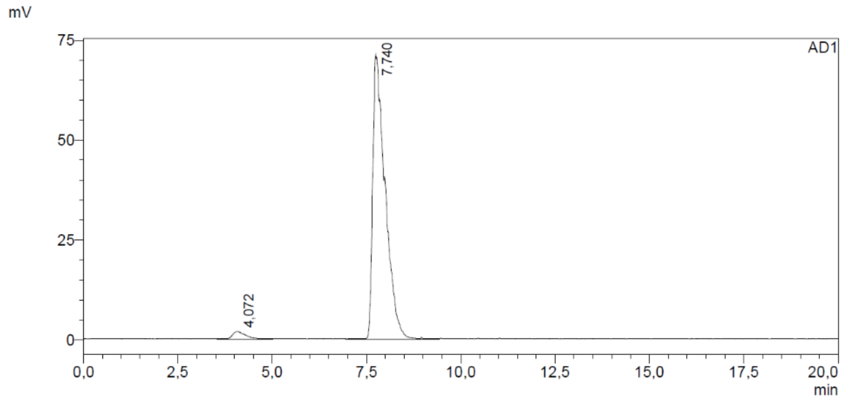

4. Transglycosylation reaction with WB + pentanol 50% + Cellic Ctec2 after 1h of reaction
   Pentyl xyloside DP1 : Rt 10.5 min
   Pentyl xyloside DP2 : Rt 8.5 min
   Pentyl xyloside DP3 : Rt 7.5 min
   D-xylose and D-glucose : co-elution at Rt 3.9 min

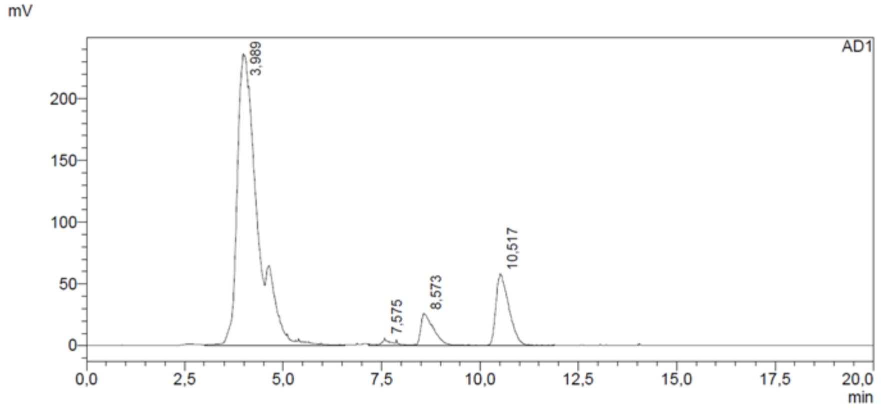

D-glucose and D-xylose laurate esters

D-glucose and D-xylose laurate esters were quantified by HPLC as reported in a previous study (Meline, Muzard et al. 2018). The quantification was performed by HPLC (Prominence, Shimadzu) using a Nucleoshell® RP 18plus 5 µm, 250 × 4.6 mm (Macherey Nagel) column at 40 °C. D-Glucose and D-xylose laurate esters were eluted at 0.8 mL/min, at 40 °C and with a 80:20 acetonitrile : water mobile phase. The detection was performed with a dynamic light scattering detector (ELSD-LT II, Shimadzu) at 40 °C under 350 kPa azote pressure.

1. D-glucose laurate monoester (standard) : Rt : 4.2 min

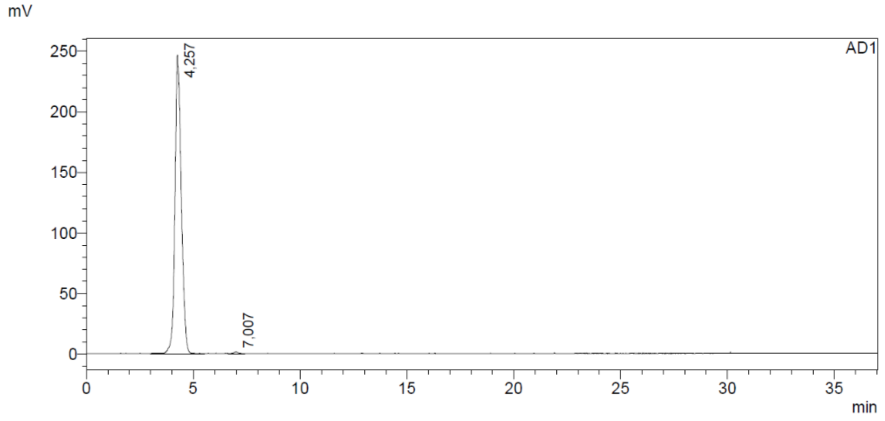

2. D-xylose laurate monoester and diesters (standard) :
   D-xylose laurate monoester : Rt 6.2 min
   D-xylose 2,5-*O*-laurate diester : Rt 28.9 min
   D-xylose 3,5-*O*-laurate diester : Rt : 30.3 min

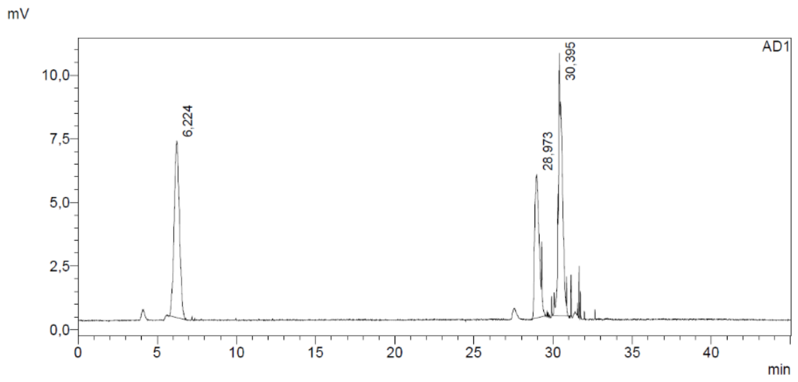

3. Esterification reaction with wheat bran hydrolysate (D-glc + D-xyl = 100 mM) + lauric acid (300 mM) + lipase N435 1% at 50°C during 48h
   D-glucose + D-xylose : co-elution at Rt 3.1 min
   D-glucose laurate monoester : Rt 4.03 min
   D-xylose laurate monoester : Rt 6.7 min

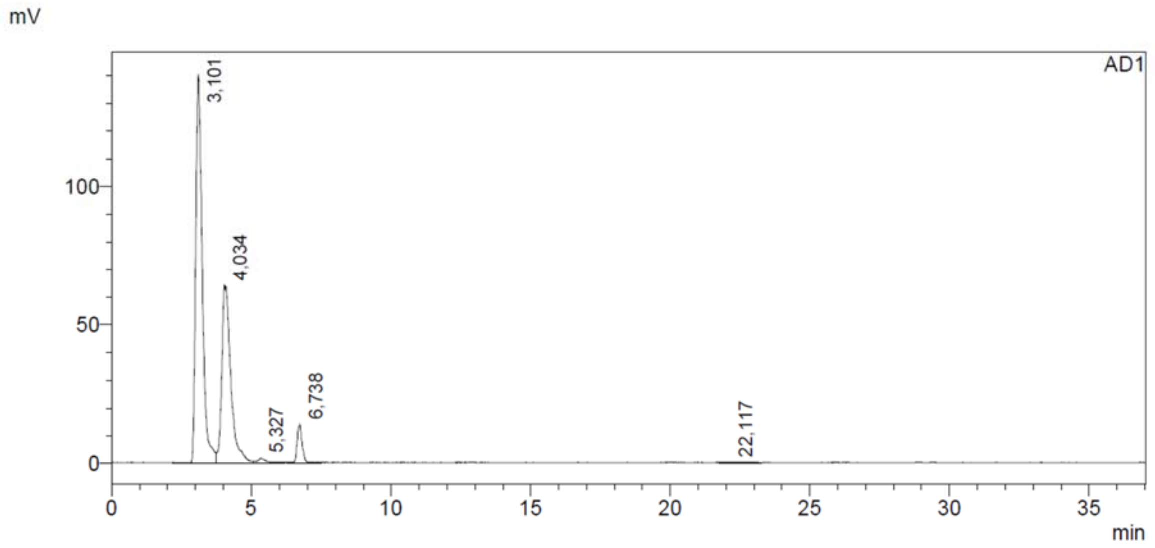

Supplement: Supplementary file 1 [file Presentation_1.pdf]
